# Quasi-experimental Study of Systematic Screening for Family Planning Services among Postpartum Women Attending Village Health and Nutrition Days in Jharkhand, India

**DOI:** 10.5334/ijic.3078

**Published:** 2018-01-25

**Authors:** Sudharsanam Balasubramaniam, Somesh Kumar, Reena Sethi, Elaine Charurat, Kamlesh Lalchandani, Anne Schuster, Bulbul Sood

**Affiliations:** 1 Jhpiego, 29 Okhla phase three, New Delhi 110020, IN; 2 Jhpiego, 1615 Thames Street, Baltimore, MD 21231, US

**Keywords:** postpartum, community, family planning, integration, immunization

## Abstract

**Background:**

Systematic screening helps increase family planning uptake through integration with other services, including immunization. Though successfully demonstrated at health facilities, this strategy has not been demonstrated in communities. This study assessed the effectiveness of systematic screening to increase postpartum family planning use during community health days in India without adversely affecting immunization services.

**Methods:**

The study was conducted during 180 individual Village Health and Nutrition Days in Jharkhand, India. All health workers were trained in postpartum family planning counseling. Intervention providers were also trained in systematic screening. 217 postpartum women aged 15–49 years participated in baseline and endline exit interviews and routine service statistics were analyzed from 2,485 facility visits at affiliated health centers.

**Results:**

No difference in family planning service use was found in the intervention group, but significantly fewer interviewed women reported receiving family planning services at endline in the comparison group (p = 0.014). Family planning acceptance at affiliated health centers increased significantly in intervention areas (p < 0.001) but not in comparison areas, while immunization service use increased in both groups (p = 0.002 intervention, p < 0.001 comparison).

**Conclusions:**

The use of the postpartum systematic screening tool appears to increase acceptance of family planning services when integrated with community-based services in Jharkhand.

## Introduction

In recent years, international health experts have paid increasing attention to improving access to and utilization of critical health services through integrated approaches, as demonstrated by the World Health Organization and the United Nations Children’s Fund identification of the integration of immunization and other health services as a key strategy for improving vaccination coverage, [1] and the United States Agency for International Development’s listing of family planning-immunization integration as a promising High Impact Practice for family planning [2]. Commitment to integration is especially high in India, which launched the comprehensive Reproductive, Maternal, Newborn, Child, and Adolescent Health Strategy in 2013. This strategy takes a life-cycle approach to women’s health and includes the integration of reproductive health services with maternal, newborn, and child health to decrease unmet need for family planning from 21% to 8.8% from 2013–2018 and to increase coverage of three doses of diphtheria-tetanus-pertussis by 3.5% annually [3].

The targeting of postpartum women through integrated service delivery is particularly important because they often have a higher unmet need for family planning, [4,5] but may not perceive themselves to be at risk because their menses have not returned and/or they are breastfeeding [6,7]. Postpartum women may not seek health services to space their next pregnancy, but are likely to seek routine immunization services for their children [8,9]. Women bringing their children to facilities according to the routine vaccination schedule have multiple contacts with health providers over the course of their first year postpartum, providing opportunity to introduce integrated messaging on a number of critical health issues, including family planning [2,10].

Strategies like systematic screening contribute to the goals set out during the 1994 Cairo International Population and Development Conference and the 2015 London Summit on Family planning, which promoted the integration of family planning into the continuum of care in order to improve access and utilization [11]. Early studies done in Togo and Mali show that integrating family planning services during the first twelve months after delivery with routine child immunization care can potentially reduce unmet need for family planning [10,12,13,14]. However, assessment of integration has focused on health facilities, despite the reliance of many women in low-resource settings on community health workers. The dearth of evidence for integration led the United States Agency for International Development’s flagship global maternal and child health program, the Maternal and Child Health Integrated Program, to evaluate approaches to integration, including the use of postpartum systematic screening during childhood immunization visits to improve access to postpartum family planning in rural India.

Systematic screening, a United States Agency for International Development best practice originally developed by Population Council, is a simple procedure that allows health care providers to reduce unmet need for health services by addressing clients’ multiple needs during a single visit. With a standardized checklist, providers first identify each client’s needs and desires for services, and then provide these services either during the same visit, or through referrals. Systematic screening has been proven successful in operations research in India and countries in Latin America and Africa. These studies found that systematic screening increases the number of services received, reduces unmet need for services, improves cost efficiency, and increases provider satisfaction [15,16]. In postpartum systematic screening, service providers utilize a checklist with particular sensitivity to postpartum women. This tool helps to identify the need for family planning along with other services specifically required during the postpartum period.

The Maternal and Child Health Integrated Program first tested the use of postpartum systematic screening to increase family planning service utilization in Nigeria [17]. The study found that among women with family planning needs, the proportion of women who received family planning counseling or were referred for additional family planning services increased significantly (17% during baseline and 68% during endline) after postpartum systematic screening was introduced [17]. Field testing in Nigeria demonstrated the potential for systematic screening to improve access to postpartum family planning at health facilities. To understand the benefits of using the postpartum systematic screening tool in a community setting, this pilot project was designed and implemented in Jharkhand, India.

Jharkhand is a comparatively small state located in eastern India, and is among the nation’s poorest with 49.6% of the population in the country’s lowest wealth quintile. Less than half, 40%, of all households have electricity, and fewer than 60% have access to improved drinking water. Jharkhand state had an overall unmet need for family planning of about 23%, among the highest state rates in India, which reported an overall unmet need for family planning of around 13% [18]. However, unmet need during the postpartum period may be much higher, with one study estimating that approximately 65% of married women between the ages of 15–49 had unmet need for family planning during the first year postpartum [4]. Despite the challenges residents face accessing many health services, Jharkhand has high rates of child immunization – at least 90% of all children in Jharkhand had received at least one vaccination – and the state has roughly the national average for fully immunized children at age two (34%) [18]. The comparatively high rates of child immunization may be due to the success of vaccination outreach campaigns in India, including the introduction of Village Health and Nutrition Days by the National Rural Health Mission [19].

In Jharkhand, Village Health and Nutrition Days take place in each community once a month on set days at health sub-centers or other suitable settings in the community. Village Health and Nutrition Days provide villagers in rural and underserved areas access to a basic package of maternal, newborn, and child health services free of charge through individual medical appointments for women and their families. Village Health and Nutrition Days also include health information sessions for groups of pregnant and lactating mothers to discuss multiple health topics, including family planning. Services are provided by a mix of clinically-trained nurse midwives, who provide primary health services such as antenatal care and immunization, and community health workers, who mobilize community members and provide counseling [20].

While a range of services are meant to be available, many families attend Village Health and Nutrition Days specifically to seek child immunization and nutritional care, and the package of other services offered at Village Health and Nutrition Days is inconsistent. For example, an assessment of the services available at 26 Village Health and Nutrition Days in Uttarakhand, in northern India, found that while nearly all surveyed Village Health and Nutrition Days (95.8%) offered vaccinations for eligible children, less than half (45.8%) offered family planning counseling and commodities [21]. In Jharkhand, a 2012 program evaluation of services provided at Village Health and Nutrition Days found that while 80% of newly delivered women received tetanus toxoid injections, only 6.2% participated in group counseling or discussions about general health issues [20]. Collectively, information from previous assessments of Village Health and Nutrition Days suggest that they offer potential for service integration, but that this has not yet been universally achieved.

A standardized checklist tool could help providers consistently integrate care by first identifying each client’s needs and desires for diverse services and then providing these services either during the same visit or through referrals. For this study, a systematic screening checklist tool with particular sensitivity to postpartum women was tested. Using a postpartum systematic screening tool during Village Health and Nutrition Days has the potential to ease service integration by introducing a structured tool to help providers and community-based health workers’ systematically identify women with unmet family planning needs and work with them to either receive a method of family planning that day, or link them to additional resources so that they can receive their preferred method. This minimizes missed opportunities to provide family planning services by working with women who have already come into contact with the health system. This study aimed to assess the effectiveness of postpartum systematic screening on increasing access to family planning services among postpartum women attending Village Health and Nutrition Days in Jharkhand state, India. The study specifically sought to determine whether availability of postpartum systematic screening (1) increases the provision of family planning counseling, methods, and referrals for postpartum women, and (2) has any effect on utilization of childhood immunizations. In order to meet these aims, the study integrated postpartum family planning with community-based immunization services during Village Health and Nutrition Days in one of 24 districts of Jharkhand state.

## Theory and Methods

### Study Overview

The study used a pre/post intervention quasi-experimental design to assess the effectiveness of the postpartum systematic screening tool on use of postpartum family planning and immunization. In consultation with the Government of Jharkhand, the Kolebira block of Simdega district in Jharkhand was selected. Simdega district has a population of approximately 600,000 people with 93% living in rural areas [22]. In 2007–2008, only 23.4% of married women of reproductive age were using any method of family planning according, with an estimated unmet need of 46.7% [23]. Most women choosing to use family planning relied on female sterilization (11.7%). In comparison, 61% of children are fully immunized [23].

Simdega district has one sub-district hospital and 155 health sub-centers. Eighteen of these sub-centers in Kolebira block were selected for this study, with a catchment population of 71,368 [24]. The catchment areas of Kolebira block’s eighteen sub-centers were randomly assigned to intervention and comparison groups, comprising nine sub-centers each. Data were collected through two sources. First, data collectors conducted exit interviews with postpartum women over 15 who were utilizing services at Village Health and Nutrition Days. The study also included longitudinal collection of service statistics from the eighteen health sub-centers hosting the Village Health and Nutrition Days.

All nurse midwives and community health workers from study sites, both intervention and comparison, participated in a 1–2 day training (depending on cadre) consisting of classroom-based and hands-on participatory exercises in postpartum family planning counseling and service delivery, and received a Contraceptive Technology Update. All providers participated in this initial training in order to assure comparable basic provider knowledge and skills across study districts. This aimed to increase the likelihood that observed changes were due to the use of the postpartum systematic screening tool rather than improved provider skills in the intervention area. Following the initial training, providers from the intervention sites received additional training in the use of the postpartum systematic screening tool. In total, nine midwives and 44 community health workers each from intervention and control sites were trained.

The postpartum systematic screening tool was used by providers enrolled in the study at the nine intervention sub-centers during contacts with postpartum women attending selected Village Health and Nutrition Days. The postpartum systematic screening tool was a bilingual (in English and Hindi) systematic screening tool administered through face-to-face client and provider interaction. The paper-based tool uses an algorithm approach through which the health workers could assess a woman’s situation and conclude if the woman needed any other services including screening of pregnancy, antenatal care, postnatal care, immunization, and postpartum family planning. Training all providers in the tool helps support universal and consistent screening for FP need across provider type and geographic location. It was field-tested in a different block of Simdega district prior to the start of data collection to ensure that it had been appropriately adapted from the Nigerian tool.

The Institutional Review Board of the Johns Hopkins Bloomberg School of Public Health approved the study protocol under IRB00003892. The government of Jharkhand state also approved the study.

### Client Exit Interviews

Data were collected from a total of 180 Village Health and Nutrition Days. At baseline, data were collected from postpartum women at a total of 60 immunization sessions, 30 each from Village Health and Nutrition Days held by the intervention and comparison sub-centers. Eligible clients were selected from the list of beneficiaries attending the session on the day of visit. Married women between 15–49 years were considered eligible if they had attended an immunization session at a selected Village Health and Nutrition Day and had delivered a child within the preceding 12 months. Women were enrolled in the study after obtaining oral informed consent and were interviewed by Program-trained data collectors. On average, two eligible clients per site were interviewed yielding exit interview data from a total of 116 women at baseline. After introducing the postpartum systematic screening tool and training providers, data was collected again from the same immunization session sites: 60 each from the intervention and control sub-centers. While the project aimed to interview two eligible clients per site for a total of 120 respondents, we were only able to interview 101 women due to incomplete data from some sub-centers. The topics for the client exit interview included client awareness and perspectives for healthy timing and spacing of pregnancy and postpartum systematic screening services, services received during the session, services for which the client was referred, and satisfaction with the visit. Data were collected between January and September, 2012.

### Service Statistics

The Program planned to use service statistics from all 180 Village Health and Nutrition Days to track the number of family planning, antenatal care, newborn care, and immunization visits and contraceptive methods distributed as part of ongoing program monitoring. Data were to be collected continuously from baseline to endline, and extracted at these points to analyze trends in service utilization over the course of the intervention. However, during start-up, the study team discovered a rudimentary routine reporting system at the facilities that was not capturing all required information. Program staff worked with the Government of Jharkhand and selected facilities to introduce a routine reporting system to the 18 health sub-centers hosting Village Health and Nutrition Days. Once this system was in place, service statistics data were collected on a continuous basis, and were abstracted at two points for analysis. However, these two points are midline and endline because service statistics were not available at baseline due to the aforementioned challenges.

### Analysis

The primary intervention outcomes, measured using client exit interview data, were the counseling, acceptance of and referral to family planning services for postpartum women, which was used as a proxy for measuring acceptance. Secondary outcomes, measured using service statistics data, included catchment area family planning acceptance and receipt of child immunization services.

Continuous variables were compared between groups using ANOVA. Categorical variables were compared between groups using chi-square tests. Dependent variables included the receipt of the third dose of diphtheria-tetanus-pertussis, which was used to measure use of immunization services. The study monitored the third dose of diphtheria-tetanus-pertussis because it is not traditionally part of mass immunization campaigns, is a good measure for immunization drop-out, and was offered as a service during Village Health and Nutrition Days [25]. All analyses were conducted using Stata 12 (Stata Corporation, College Station, TX), and Epi Info 7.1.6.0 (CDC, Atlanta, GA) with a type I error of 0.05.

## Results

### Exit Interviews

In total, 217 clients were interviewed in 30 sites, with approximately 55 clients in each group. Women’s demographic characteristics did not differ significantly between the intervention and comparison groups or between baseline and endline (Table 1). Participants were an average of 26 years old with two living children. Most women (>90%) were either Christian or Hindu. More than half of all women were living below the poverty line. Only about one third of women had completed secondary level education.

**Table 1:** Demographic characteristics of postpartum women visiting Village Health and Nutrition Days at baseline and endline in Kolebira block, Simdega district, Jharkhand, India (N = 212^*^).

Demographic characteristic^1^		Baseline		Endline	

		Systematic Screening (n = 56)	Comparison (n = 59)	Systematic Screening (n = 48)	Comparison (n = 49)

Age, years		26.6 (5.3)	26.2 (4.3)	26.6 (5.2)	27 (4.4)
Religion (%)	Christian^2^	37 (66.1)	27 (45.8)	30 (57.7)	21 (42.0)
	Hindu	19 (33.9)	27 (45.8)	20 (38.5)	27 (54.0)
	Others	0	5 (8.5)	2 (3.9)	2 (4.0)
Education (%)	No schooling	20 (35.7)	19 (32.2)	24 (47.1)	16 (32.7)
	Primary	19 (33.9)	23 (39.0)	13 (25.5)	17 (34.7)
	Secondary	15 (26.8)	10 (17.0)	12 (23.5)	11 (22.5)
	Higher	2 (3.6)	7 (11.9)	2 (3.9)	5 (10.2)
Socio economic status (%)	Below poverty line	34 (60.7)	33 (55.9)	31 (64.6)	31 (62.0)
	Above poverty line	22 (39.3)	26 (44.1)	17 (35.4)	19 (38.0)
	Total Children	2.4 (1.2)	2.3 (1.3)	2.3 (1.0)	2.2 (1.1)

^*^ Missing data for 5 respondents. ^1^ Means (SD) for continuous variables with normal distribution, % for categorical variables. ^2^ A significant difference was noted between the number of Christian respondents in intervention and control groups at baseline. The difference was not significant at endline, or from baseline to endline in either intervention or control groups.

Based on exit interviews, the majority of women (70.4% at baseline and 89.2% at endline) attended the Village Health and Nutrition Day for child immunization services. As Table 2 shows, significantly more women at endline stated that they visited the Village Health and Nutrition Day for immunization services compared to baseline in both intervention (p = 0.0357) and comparison (p = 0.0032) groups.

**Table 2:** Women’s reasons for visiting Village Health and Nutrition Days in intervention and comparison groups in Kolebira block, Simdega district, Jharkhand, India (N = 212^*^).

	Type of service	Baseline (n = 54)	Endline (n = 50)	*P*^3^

Intervention	Newborn Care	7 (13%)	3 (6%)	0.3839
	Child Immunization	40 (74%)	45 (90%)	0.0357
	Sick Baby	2 (4%)	0 (0%)	0.5095
	Other Service	5 (9%)	2 (4%)	0.4981
	**Type of Service**	**Baseline (n = 59)**	**Endline (n = 49)**	***P***

Comparison	Newborn Care	13 (22%)	3 (6%)	0.0408
	Child Immunization	41 (70%)	46 (96%)	0.0032
	Sick Baby	1 (2%)	0 (0%)	0.9244
	Other Service	4 (7%)	0 (0%)	0.1783

^*^ Missing data for 5 respondents. ^3^ Yates’ Chi-Square used with cells fewer than 5.

None reported coming to the Village Health and Nutrition Day for family planning services (data not shown). However, more than half of the women reported that they received family planning services (defined as counseling, acceptance, or referral) as a secondary service during their visit (Table 3).

**Table 3:** Receipt of family planning services by women visiting Village Health and Nutrition Days in Kolebira block, Simdega district, Jharkhand, India (N = 217).

	Type of service	Baseline (n = 56)	Endline (n = 52)	*P*

Intervention	Family planning Counseling	28 (50.0%)	22 (42.3%)	0.423
	Family planning Acceptantenatal caree or Referral	9 (16.1%)	12 (23.1%)	0.358
	Any Family planning Provided	37 (66.1%)	34 (65.4%)	0.938
	**Type of Service**	**Baseline (n = 59)**	**Endline (n = 50)**	***P***

Comparison	Family planning Counseling	38 (64.4%)	22 (44.9%)	0.042
	Family planning Acceptantenatal caree or Referral	15 (25.4%)	13 (26.5%)	0.896
	Any Family planning Provided	53 (89.9%)	36 (72.0%)	0.014

The percent of women who reported receiving family planning services did not change in the intervention group during the project period (66.1% vs. 65.4%). In the comparison group, significantly fewer women (89.9% vs. 72%, p = 0.014) reported receiving family planning services at endline. Table 3 breaks this information down by type of family planning service: counseling and method acceptance or referral. The data shows that while overall services decreased among both intervention and comparison groups, acceptance of a method or referral increased: from 16.1% to 23.1% in the intervention group and from 25.4% to 26.5% in the comparison group. However, this relationship was not significant for either group.

Women were given the opportunity to answer an open-ended question about their decision to either accept or not accept a method of family planning. Most women (82.9%) did not respond. However, among those providing a reason, responses were varied, but common reasons for not accepting a method included fear, money problems or lack of money, inability to leave the home for follow-up, and objections from other family members. The most common response was fear, which approximately 15% of women answering the questions cited as a reason for not accepting a method. The sample of respondents to this question is too small to conduct further analysis.

### Service Statistics

Longitudinal service statistics from 3,035 postpartum women attending 18 study facilities linked to the observed Village Health and Nutrition Days were reviewed. Demographic information about the clients was not available, as this information is not collected at the facilities. Data show that the number of children receiving the third dose of diphtheria-tetanus-pertussis increased significantly from 19.3% to 27.1% in the intervention (p = 0.002) and from 14.5% to 24.4% in the comparison areas (p < 0.001) during the study period (Table 4). This data also showed that distribution of family planning methods significantly increased from 34.4% to 53.5% (p < 0.001) in the intervention group but did not change in the control group, 35.2% to 36.3% (p = 0.667).

**Table 4:** Family planning and immunization service use according to service statistics from Kolebira block, Simdega district, Jharkhand, India.

	Type of service	Midline (n = 378)	Endline (n = 1220)	*P*

Intervention	Family planning acceptantenatal caree	130 (34.4%)	653 (53.5%)	<0.001
	DPT immunization given to child	73 (19.3%)	330 (27.1%)	0.002
	**Type of Service**	**Midline (n = 613)**	**Endline (n = 824)**	***P***

Comparison	Family planning acceptantenatal caree	216 (35.2%)	299 (36.3%)	0.667
	DPT immunization given to child	89 (14.5%)	201 (24.4%)	<0.001

## Discussion

This study was initiated during scheduled health outreach events in rural communities in India to assess whether the systematic screening tool could increase the proportion of postpartum women being referred to or accepting family planning services. Results of the study, measured by service statistics and client exit interviews, show a statistically signficant increase in family planning acceptantence and referrals at Village Health and Nutrition Days with a concommitant increase in immunization services in the intervention as compared to the control sites.

The increased acceptance of family planning found by examining the service statistics of the present study is consistent with findings from examinations of the use of systematic screening in urban clinics and health sub-centers in India and of the postpartum systematic screening tool in Nigeria [15,17]. Compared to the previous study in urban India, the results from this study showed a lower acceptance of family planning, 53.5% compared with more than 90%. However, both programs were linked to higher family planning utilization than in India overall (23.4%) which is normally lower in rural areas [23].

Although the experience in Jharkhand shows the potential for integrating services using systematic screening at the community level, the program and study faced several challenges. Programmatic results suggest that despite using the postpartum systematic screening tool during Village Health and Nutrition Days, tracking receipt of long-acting methods remained a challenge as these were not available on site. Client exit interviews and service statistics both suggest an increase in utilization of family planning. However, in cases where women were interested in long-acting methods, they were referred to facilities for provision of the method. The program and study teams did not have a system in place to monitor where women were referred to or to confirm that referred women ultimately received family planning. However, service statistics from affiliated facilities did show a significant increase in the distribution of family planning methods over the study period in areas where Postpartum Systematic Screening was used. Similar trends were not observed at facilities located near Village Health and Nutrition Days that provided routine care without the use of the Postpartum Systematic Screening tool. While service statistics suggest that method distribution may have increased as a result of the systematic screening tool, the relationship cannot be statistically demonstrated, a challenge noted in other studies evaluating systematic screening,[16,17].

Furthermore, the study was challenged in the concurrent monitoring of immunization services, a challenge previously noted in several studies on family planning-immunization integration in Africa [10,13,14,26]. Steps should be taken in future research and programming to address these challenges.

The results are also limited due to the small sample size, which was too small to capture the lower than expected percent change in postpartum family planning acceptance captured by the program (a 50% increase in family planning utilization was expected, but only an approximately 6% increase was observed). The small sample size was in part due to a higher than anticipated non-response rate, limiting the effect that could be measured. While a certain level of bias is expected in client exit interviewers, this study may have higher than normal response bias as interviews were completed by male data collectors due to the difficult local terrain and cultural practices that limited the ability to hire female data collectors.

Additionally, collection of service statistics data was limited due to existing systematic barriers, namely the limited functionality of health information systems at baseline. Through working with local health officials, the Program was able to strengthen the routine reporting system; however, because of this unanticipated need, the effect of postpartum systematic screening on services, as measured by service statistics, could only be assessed at midline and endline. As a result, we cannot describe the full potential of the postpartum systematic screening on either family planning or immunization service delivery. However, considering the midline to endline changes in service statistics, we assume that the overall effect would have been greater if a comparison to baseline numbers could be made. Given the use of a quasi-experimental design, the lack of other interventions in the said period, and the comparison group’s lack of a significant increase in uptake of family planning services, we surmise that the difference in family planning uptake may be due to use of postpartum systematic screening in the intervention Village Health and Nutrition Days.

Future research should consider evaluating the cost-effectiveness of postpartum systematic screening in similar, rural, community-based health service delivery settings, the extent of the burden that using the tool may place on service providers and/or community health workers, and ways to streamline the process of integrating health services. Further work is needed to standardize use of the tool across the state and to develop a more robust system for providing support to providers for training and continuing education. Despite these limitations, the data suggest that systematic screening could be used to integrate postpartum family planning services without affecting the immunization services in Jharkhand and perhaps other similar settings in India.

## References

[1] Global immunization vision and strategy 2006–2015. New York City: Unicef; 10 2005 Available from: http://apps.who.int/iris/bitstream/10665/69146/1/WHO_IVB_05.05.pdf.

[2] High-Impact Practices in Family planning (HIP). Family planning and immunization integration: reaching postpartum women with family planning services Washington, DC: USAID; 2013 7 Available from: http://www.Familyplanninghighimpactpractices.org/resources/family-planning-and-immunization-integration-reaching-postpartum-women-family-planning.

[3] Government of India, Ministry of Health and Family Welfare. A strategic approach to reproductive, maternal, newborn, child and adolescent health (RMNCH+A) in India New Delhi: Ministry of Health and Family Welfare, 1, 2013 Available from: http://nrhm.gov.in/images/pdf/RMNCH+A/RMNCH+A_Strategy.pdf.

[4] Family planning needs during the extended postpartum period in India. Washington (DC): ACCESS-FAMILY PLANNING; 2009 ACCESS-FAMILY PLANNING Initiative Brief. Available from: http://pdf.usaid.gov/pdf_docs/pnaea344.pdf.

[5] Ross, J and Winfrey, W. Contraceptive use, intention to use and unmet needs during the extended postpartum period. International Family planning Perspectives, 2001; 27(1): 20–27. DOI: 10.2307/2673801

[6] Cleland, J, Shah, I and Benova, L. A fresh look at the level of unmet need for family planning in the postpartum period, its causes and program implications. Int Perspect Sex and Reprod Health, 2015; 41(3): 155–162. DOI: 10.1363/intsexrephea.41.3.155 26600569

[7] Moore, Z, Pfitzer, A, Gubin, R, et al. Missed opportunities for family planning: an analysis of pregnantenatal carey risk and contraceptive method use among postpartum women in 21 low- and middle-income countries. Contraception, 2015; 92: 31–39. DOI: 10.1016/j.contraception.2015.03.007 25769442 10.1016/j.contraception.2015.03.007

[8] Cleland, J, Conde-Agudelo, A, Peterson, H, et al. Contraception and health. Lantenatal careet, 2012; 380: 149–56. DOI: 10.1016/S0140-6736(12)60609-6 22784533

[9] Huntington, D and Aplogan, A. The integration of family planning and childhood immunization services in Togo. Stud Fam Plann, 1993; 25(3): 176–83. DOI: 10.2307/2137943 7940622

[10] FHI 360/PROGRESS. Postpartum family planning: New research findings and program implications. 2012b Available from: http://www.fhi360.org/sites/default/files/media/documents/Postpartum%20Family%20Planning.pdf.

[11] London summit on family planning: executive summary. Family planning 2020, 7 11, 2012 Available from: http://ec2-54-210-230-186.compute-1.amazonaws.com/wp-content/uploads/2013/09/London-Summit-Family-PlanningOverview_V1-14June.pdf.

[12] Population Services International. ProFam urban outreach: A high impact model for family planning 2012; Washington, DC: PSI.

[13] Cooper, CM, Fields, R, Mazzeo, CI, et al. Successful proof of concept of family planning and immunization integration in Liberia. Global Health Sci Pract, 2015; 3(1): 71–84. DOI: 10.9745/GHSP-D-14-00156 PMC4356276 25745121

[14] Vantenatal caree, G, Janowitz, B, Chen, M, et al. Integrating family planning messages into immunization services: a cluster-randomized trial in Ghana and Zambia. Health Policy Plan, 2014; 29(4): 359–366. 23570834 10.1093/heapol/czt022

[15] Das, NP, Shah, U, Chitania, V, et al. Systematic screening to integrate reproductive health services in India Vadadora: Population Council; 2005 FRONTIERS study report. Available from: http://www.popcouncil.org/uploads/pdfs/frontiers/FR_FinalReports/India_Screen.pdf.

[16] Foreit, JR. Systematic screening: a strategy for determining and meeting clients’ reproductive health needs Washington, D.C., Population Council, Frontiers in Reproductive Health, 2006 3 8 Available from: http://pdf.usaid.gov/pdf_docs/pnaec204.pdf.

[17] Charurat, E, Nasir, B, Regina Airede, L, et al. Postpartum systematic screening in northern Nigeria: A practical application of family planning and maternal newborn and child health integration Washington (DC): ACCESS-FAMILY PLANNING; 2010 ACCESS-FAMILY PLANNING Technical Report.

[18] National family health survey, India, 2005–06. Mumbai: International Institute for Population Sciences and Macro International; 2007 Available from: http://www.rchiips.org/NFHS/NFHS-3%20Data/VOL-1/India_volume_I_corrected_17oct08.pdf.

[19] Partapuri, T, Steinglass, R and Sequeira, J. Integrated delivery of health services during outreach visits: A literature review of program experience through a routine immunization lens. J Infect Dis, 2012; 205(sup 1): S20–S27. 22315382 10.1093/infdis/jir771 PMC3273971

[20] IntraHealth International. Improving the coverage and quality of Village Health and Nutrition Days Chapel Hill (NC): IntraHealth International; 10 2012 Available from: http://www.intrahealth.org/files/media/improving-the-coverage-and-quality-of-village-health-and-nutrition-days/VILLAGEHEALTHANDNUTRITIONDAY_UP_30_10_12.pdf.

[21] Saxena, V, Kumar, P, Kumari, R, et al. Availability of village health and nutrition day services in Utterakhand, India. J Fam Med Prim Care, 2015; 4(2): 251–256. DOI: 10.4103/2249-4863.154667 PMC4408710 25949976

[22] Government of India, Ministry of Home Affairs, Registrar General & Census Commissioner. Block wise primary census abstract data (PCA) – Jharkhand. 2011 Census New Delhi: Registrar General & Census Commissioner, 2011 Available from: http://www.censusindia.gov.in/2011census/population_enumeration.html.

[23] Government of India, Ministry of Health and Family Welfare. District level household and facility survey, 2007–08 Mumbai: International Institute for Population Sciences; 2008 Available from: http://www.jsk.gov.in/dlhs3/India.pdf.

[24] District Administration, Simdega. District at a Glantenatal caree. Available from: http://simdega.nic.in/profile.html.

[25] Bos, E and Batson, A. Using immunization coverage rates for monitoring health sector performantenatal caree: measurement and interpretation issues Washington (DC): The International Bank for Reconstruction and Development/The World Bank; 2000.

[26] Research findings: Integration of postpartum family planning with child immunization in Rwanda. Research Triangle Park (NC): FHI 360; 8 2013.

